# A Comprehensive Review on Minimally Destructive Quality and Safety Assessment of Agri-Food Products: Chemometrics-Coupled Mid-Infrared Spectroscopy

**DOI:** 10.3390/foods14223805

**Published:** 2025-11-07

**Authors:** Lakshmi B. Keithellakpam, Renan Danielski, Chandra B. Singh, Digvir S. Jayas, Chithra Karunakaran

**Affiliations:** 1 Department of Biosystems Engineering, University of Manitoba, Winnipeg, MB R3T 5V6, Canada; keithelb@myumanitoba.ca (L.B.K.); chithra.karunakaran@lightsource.ca (C.K.); 2 Advanced Post-Harvest Technology Centre, Lethbridge Polytechnic, 3000 College Drive S., Lethbridge, AB T1K 1L6, Canada; renan.danielski@lethpolytech.ca; 3 President’s Office, University of Lethbridge, Lethbridge, AB T1K 3M4, Canada; 4 Canadian Light Source, Saskatoon, SK S7N 2V3, Canada

**Keywords:** FTIR, microbial contamination, chemometrics, food quality, safety, non-destructive

## Abstract

Ensuring the quality and safety of agricultural and food products is crucial for protecting consumer health, meeting market expectations, and complying with regulatory requirements. Quality and safety parameters are commonly assessed using chemical and microbiological analyses, which are time-consuming, impractical, and involve the use of toxic solvents, often disrupting the material’s original structure. An alternative technique, infrared spectroscopy, including near-infrared (NIR), mid-infrared (MIR), and short-wave infrared (SWIR), has emerged as a rapid, powerful, and minimally destructive technique for evaluating the quality and safety of food and agricultural products. This review focuses on discussing MIR spectroscopy, particularly Fourier transform infrared (FTIR) techniques, with emphasis on the attenuated total reflectance (ATR) measurement mode (globar infrared light source is commonly used) and on the use of synchrotron radiation (SR) as an alternative high-brightness light source. Both approaches enable the extraction of detailed spectral data related to molecular and functional attributes concerning quality and safety, thereby facilitating the assessment of crop disorders, food chemical composition, microbial contamination (e.g., mycotoxins, bacteria), and the detection of food adulterants, among several other applications. In combination with advanced chemometric techniques, FTIR spectroscopy, whether employing ATR as a measurement mode or SR as a high-brightness light source, is a powerful analytical tool for classification based on attributes, variety, nutritional and geographical origins, with or without minimal sample preparation, no chemical use, and short analysis time. However, limitations exist regarding calibrations, validations, and accessibility. The objective of this review is to address recent technological advancements and existing constraints of FTIR conducted in ATR mode and using SR as a light source (not necessarily in combination). It defines potential pathways for the comprehensive integration of FTIR and chemometrics for real-time quality and safety monitoring systems into the global food supply chain.

## 1. Introduction

The quality and safety of the food we eat are of critical importance. Food safety regulatory agencies worldwide are responsible for identifying potential hazards along the food chain and establishing and enforcing permitted limits. In recent years, concerns about food safety have been increasing. According to the Food and Agriculture Organization (FAO), around 25% of agricultural products worldwide are estimated to be contaminated by mycotoxins, fungi-produced toxins that can cause diseases and lead to death [1]. Food, whether processed or raw, must be analyzed for its quality and safety attributes. Global agriculture is under increasing pressure to provide high-quality food efficiently and responsibly, while also responding to climate change [2]. Food quality and safety must be ensured throughout the entire food chain to safeguard public health, meet legal standards, guarantee consumer satisfaction, and maintain economic viability. Food safety is crucial in preventing foodborne illnesses caused by harmful microorganisms, toxins, or chemical contaminants, which can lead to severe health problems and even fatal consequences [3]. Another concern within the realm of food safety includes food adulteration, characterized by the incorporation of hazardous, inferior, unwanted materials or unapproved chemicals into food products. Frequently motivated by monetary benefits, common adulterants include the addition of water to milk, starch to spices, and synthetic colors to sweets. These activities undermine nutritional quality and may lead to significant health hazards, including toxicity, chronic disease, and even death. Adulteration poses physical, chemical, and biological risks; therefore, ensuring food safety requires rigorous regulatory enforcement, advanced detection technology, and heightened consumer awareness. Addressing adulteration is crucial for protecting public health and preserving confidence in the food supply [4]. Conventional analytical methods tend to be slow, destructive to the sample, and less efficient; however, minimally destructive techniques, such as MIR spectroscopy, provide rapid and precise detection of adulterants and hazards, thereby enhancing the quality of foods and the safety of consumers. [5].

Spectroscopy involves the generation, measurement, and interpretation of spectra resulting from the interaction of electromagnetic radiation with matter. Spectroscopic methods are informative and widely used for both quantitative and qualitative analyses [6,7,8]. Spectroscopic techniques have emerged as important alternatives to conventional chemical analyses, particularly in the infrared (IR) region. The IR spectroscopy identifies and characterizes chemical compounds based on their absorption of infrared radiation, a type of electromagnetic wave with wavelengths longer than visible light but shorter than microwaves, typically experienced as heat. These spectroscopy methods yield molecular signatures of food constituents by assessing vibrational transitions of chemical bonds. The IR spectrum is typically divided into three regions, i.e., near-infrared (12500–4000 cm^−1^), mid-infrared (4000–400 cm^−1^), and far-infrared (400–10 cm^−1^) [9].

MIR (4000–400 cm^−1^) is commonly used for studying molecular vibrations. The region between 4000 and 1500 cm^−1^ is used to identify functional groups, while the 1500–600 cm^−1^ region, known as the fingerprint region, offers a unique spectral pattern for each compound. Such specificity creates a molecular fingerprint for each compound, facilitating detection. Mid-IR spectroscopy finds broad applications in food quality analysis, pharmaceuticals, environmental monitoring, medicine, and materials science due to its speed, minimally destructive nature, and the detailed chemical information it provides [10,11,12,13,14]. The spectra produced by MIR spectroscopy identify a sample’s constituents by analyzing the spectral peak location, height, and width, and are widely used to assess food lipids, proteins, and carbohydrates. It also tracks changes during processing and storage and enhances the classification and prediction of food authenticity and nutritional content using chemometrics. The combination of MIR spectrometry with chemometrics provides a comprehensive method for identifying adulterants, evaluating compositions, and monitoring deterioration or contamination with exceptional sensitivity and minimal sample preparation requirements [4,15,16,17,18,19,20]. Fourier transform infrared (FTIR) spectroscopy (MIR region, 4000–400 cm^−1^) is widely used to study molecular vibrations of food components for the detection of contaminants, adulterants (such as milk, butter, and oil), and quality assessment. FTIR has some benefits over other IR spectroscopies, such as minimal sample preparation, quick spectral data acquisition, high throughput, and avoidance of toxic chemicals. The coupling of chemometrics such as principal component analysis (PCA), partial least squares (PLS), and linear discriminant analysis (LDA) can extract valuable information from the spectral data [21,22,23].

This review provides an overview of commonly used attenuated total reflectance-fourier transform infrared (ATR-FTIR), the globar infrared light source, mostly and synchrotron (SR)-based FTIR (with and without the ATR accessory) spectrometric techniques, compiling the major parameters used for the identification of food components. Additionally, the applications of such techniques in various contexts within the agri-food sector are discussed, based on recent literature.

## 2. Attenuated Total Reflectance–Fourier Transform Infrared (ATR-FTIR) Spectroscopy

**Figure 1:**
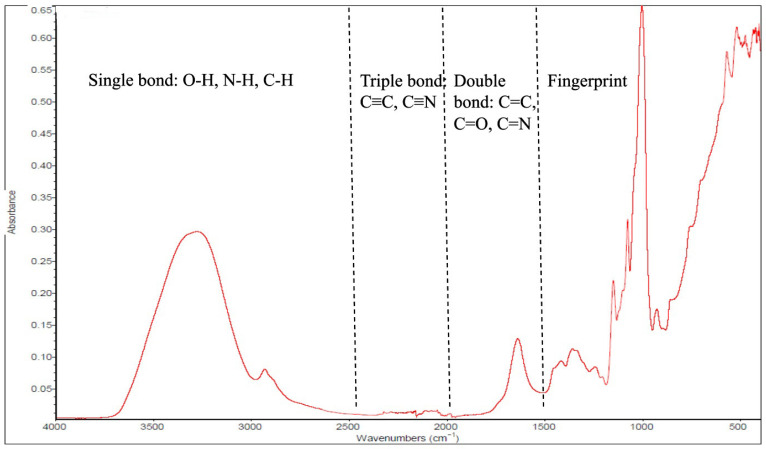
Schematic diagram of an attenuated total reflectance–Fourier transform infrared (ATR-FTIR) spectrum.

The FTIR conducted in ATR measured mode using a globar light source (ATR-FTIR) is the most common type of infrared spectrometer, providing a fast and minimally destructive method for food authentication and quantitative analysis [24]. The food sample either absorbs or transmits mid-infrared, with spectral data collected in the wavenumber range from 4000 to 400 cm^−1^ [25]. This technique has been used to analyze samples in many fields, including gynecological [26,27], solid-waste management [28], health research [29,30], textiles [31], and the quality and safety of foods (dairy, palm oil, honey, garlic) [32]. The MIR spectrum consists of two main regions: the functional group region (4000–1500 cm^−1^), which aids in identifying specific functional groups, and the fingerprint region (1500–600 cm^−1^), a complex yet unique area for each molecule that facilitates confirmation of matches with reference spectra. It can be subdivided into the single-bond region (4000–2500 cm^−1^), triple-bond region (2500–2000 cm^−1^), double-bond region (2000–1500 cm^−1^), and the fingerprint region (1500–600 cm^−1^) [33]. In the functional group region, important peaks include wide O–H (3200 and 3600 cm^−1^), N–H (3300 to 3500 cm^−1^), C–H (2850 to 2960 cm^−1^), C=O (1650 to 1750 cm^−1^), and C=C (1600 to 1680 cm^−1^) [34,35]. The ATR-FTIR spectrum has wavenumbers (cm^−1^) on the X-axis, ranging from 4000 to 400 cm^−1^, and the Y-axis indicates the amount of infrared light absorbed or transmitted at each wavenumber. Figure 1 displays a schematic ATR-FTIR spectrum. The ATR-FTIR spectroscopy enables rapid analysis without requiring complex sample preparation; however, it is essential to carefully examine the positions, shapes, and intensities of the peaks and compare them to reference data for accurate identification.

### 2.1. Principle and Instrumentation

The FTIR spectroscopic technique enables quick data acquisition with limited sample preparation and reduced sample volume requirements. Smith [36] explained that in ATR-FTIR spectroscopy, wavelength (measured in micrometres) represents the distance between wave peaks, while wavenumber (in cm^−1^) indicates the number of wave cycles that fit into one centimetre and is directly related to energy. Wavenumbers help us understand how molecules vibrate; therefore, ATR-FTIR spectra are typically displayed in wavenumbers, facilitating the identification of specific functional group absorptions. A key point about wavenumber (*W*) is that it directly relates to light energy, as shown by Equation (1) [36]. As energy increases with wavenumber, light of higher wavenumber possesses greater energy than light of lower wavenumber. Thus, the X-axis of an ATR-FTIR spectrum represents an energy scale, with higher energy values on the left and lower energy values on the right. Mid-IR spectral regions can be used to indicate the presence of major classes of food molecules, with broad absorption bands typically representing lipids, proteins, and polysaccharides found within the ranges of 3000–2800 cm^−1^, 1800–1500 cm^−1^, and 1200–900 cm^−1^, respectively. In addition to these general ranges, narrower and more specific absorption bands have been reported, serving as distinct fingerprint regions for identifying molecular components in food systems [37].

The number of scans and resolutions are critical factors that affect spectral quality. Increasing the number of scans enhances the signal-to-noise ratio (SNR), resulting in smoother and cleaner spectra; however, it also prolongs analysis time. The SNR is proportional to the square root of observation time (t; Equation (2)) [36]. The observation time is dependent on the number of scans (*N*′; Equation (3)) [36]. On the other hand, better resolution (indicated by smaller cm^−1^ values) helps to distinguish nearby peaks clearly, revealing more complex structural details. However, longer measurement times are required for achieving better resolution, which in turn produces larger datasets. Thus, optimization of both scan number and resolution is crucial for acquiring precise, high-quality spectra.

(1) E=hcW

where *E* = light energy in joules, *h* = Planck’s constant (6.63 × 10^−34^ J·s), *c* = speed of light (about 3 × 10^10^ cm/s), and *W* = wavenumber (cm^−1^).

(2) $$SNR\propto t^{1/2}$$

(3) $$SNR\;\propto \;{N\prime }^{1/2}$$

where *SNR* = signal-to-noise ratio; t = observation time; and *N*′ = number of scans.

**Figure 2:**
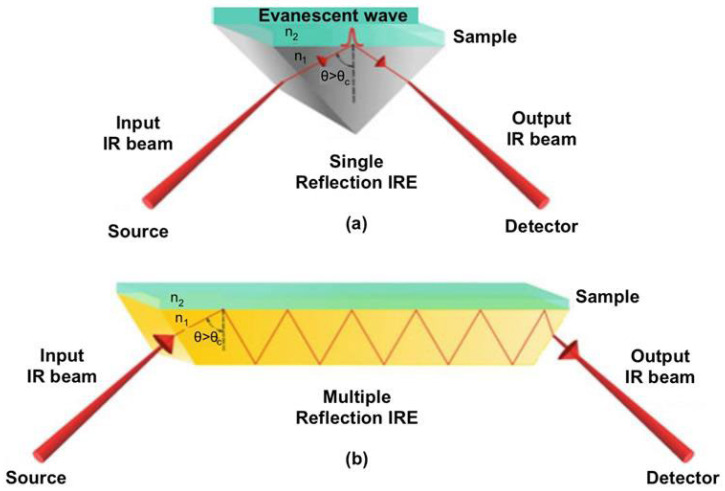
Attenuated total reflectance–Fourier transform infrared (ATR-FTIR) spectrometer optical geometries: (**a**) single and (**b**) multiple internal reflection elements (IREs). Reproduced with permission [40].

Ahmad and Ayub [38] described that most infrared sources are hot-body emitters, generally made from resilient materials heated by electric current. With increasing temperatures, both the intensity and peak wavenumber of emission increase. Common mid-infrared sources are silicon carbide rods (Globar), Nernst glowers, and tungsten glowers, while the source for the SR-based FTIR discussed in Section 3 uses synchrotron radiation [39] instead. Globar, the most prevalent source, functions at approximately 1300 K (Kelvin) and consists of a heated silicon carbide rod (2–5 cm in length, 0.5–1 cm in width), generating powerful infrared radiation. Nernst glowers, composed of refractory oxides such as cerium, zirconium, and thorium, can attain temperatures between 1500 and 2000 K and exhibit efficiency below 2000 cm^−1^, while their effectiveness diminishes at higher wavenumbers. Quartz–tungsten–halogen lamps, which feature inert gas and halogen, produce tungsten oxy-halide, thereby extending lamp longevity. ATR-FTIR spectroscopy assesses samples with a penetration depth ranging from 0.5 to 5 μm from the sample surface. ATR-FTIR spectroscopy utilizes a specialized component known as the internal reflection element (IRE), which features a high refractive index (n1) in conjunction with a non-transparent sample (refractive index, n2). The IRE commonly comprises diamond, silicon, zinc selenide, or germanium. When selecting the crystal or IRE, the sample’s sensitivity level, pH, toughness, resilience, and overall size are considered. Internal reflection may occur via single or multiple reflection geometries, facilitated by the diverse geometries of internal reflection elements (IRE) employed in the ATR system. Importantly, the ATR accessory is not limited to FTIR systems operating with the Globar infrared source. The ATR can also be coupled to FTIR equipment using other light sources, such as synchrotron radiation [39]. Figure 2a,b present the optical configurations of the two geometries [40].

When the infrared beam is directed above the critical angle (*θ_c_*), total internal reflection occurs, generating an evanescent wave of radiation. The wave interacts with the sample, attenuating the IR beam as it exits the IRE. The attenuated infrared beam leaves the infrared reflective element and enters the detector, where it is transformed into an infrared spectrum. The penetration depth (*dp*) depends on the wavelength and refractive indices as given in Equation (4) [41].

(4) $$dp=\frac{\lambda }{2\pi n1\sqrt{{Sin}^{2\;}\theta }-{\left(\frac{n1}{n2}\right)}^{2}}$$

where *dp* = penetration depth (µm); λ = wavelength; *n*1 = refractive index of the crystal; *n*2 = sample refractive index, and *θ* = internal reflectance angle.

### 2.2. Applications of ATR-FTIR in Agri-Food Products Validation and Compositional Analysis

ATR-FTIR can be utilized in a range of applications to assess the post-harvest quality of agricultural products and the quality parameters of food products. Some of these applications include the quantification of sugar and organic acids in fresh fruits, classification of fruits according to cultivar and ripening states, cell wall composition, detection of infection-induced physical damage, assessment of flavor compounds, pigments, and antioxidant compounds, among others. The technique also enables the establishment of a compositional identity for each food, allowing for the detection of adulteration. By comparing the spectral patterns of authentic and adulterated foods, significant changes can be detected. Spectral data, when combined with chemometric techniques, can create a validated model for wider analysis. This section specifies the applications in fruits, vegetables, grains, and other crops, based on the recent literature.

#### 2.2.1. Fruits and Vegetables

The ATR-FTIR technique has become a potential approach for monitoring the quality and composition of fruits and vegetables in a minimally destructive manner. Bureau et al. [42] studied the feasibility of ATR-FTIR for sugar and organic acids in apricot slurries in the MIR range 1500–900 cm^−1^, along with the partial least squares (PLS). High predictive accuracy with R^2^ of 0.85 (sucrose), 0.87 (glucose), 0.96 (citric acid), and 0.97 (malic acid) was obtained. However, the accuracy was lower for fructose (R^2^ = 0.74). Beyond quantification, PCA revealed the method’s ability to distinguish cultivars and ripening stages, though fruit softening remained less predictable. In another study, Quaabou et al. [43] assessed phenolic acids, flavonoids, anthocyanins, and microbiological quality of pasteurized cherry syrup under varying storage conditions (temperature and duration). The ATR-FTIR technique was employed to analyze the changes in functional composition and microbial quality (yeast and mold) throughout the storage period. The spectroscopic analysis offered valuable insights into the chemical alterations associated with proteins and polyphenols. However, the study overlooked the alteration in sensory qualities, particularly the colour related to anthocyanins after storage. The study concentrated solely on yeast and mold, neglecting other species, particularly bacteria. This oversight signifies a gap or limitation in the study. Expanding the scope, Canteri et al. [44] applied ATR-FTIR to alcohol-insoluble solids (AISs) from 29 fruit and vegetable species, achieving excellent AIS yield prediction accuracy (R^2^ = 0.92). PCA segregated the samples according to their compositions. However, powdered raw materials posed challenges due to spectral interference from soluble substances. In tomatoes, ATR-FTIR combined with chemometrics has proven to be exceptionally versatile, both for compositional analysis and for the detection of diseases. Lv et al. [17] employed multivariate models to evaluate soluble sugars, achieving a high prediction accuracy of R^2^ = 0.86. The samples were then classified into high- and low-sugar groups with an accuracy of over 92%. This demonstrates its value for rapid, non-destructive discrimination of tomatoes based on sugar profiling. Extending the study from compositional quality to the detection of tomato disease, particularly sour rot, Skolik et al. [45] found that spectral fingerprints, analyzed by PCA and linear discriminant analysis (LDA), could effectively differentiate between healthy, mechanically damaged, and infected tomatoes well before visual symptoms appeared. Likewise, Vermeir et al. [46] further advanced tomato quality assessment by extracting juice from the whole tomato and integrating sequential injection analysis (SIA) with ATR-FTIR, enhancing automation and reproducibility. Their partial least squares-discriminant analysis (PLS-DA) models classified cultivars based on sugar and acid profiles, although the prediction of malic acid remained limited due to its low concentration. These studies highlight ATR-FTIR as a versatile, fast, and minimally destructive technique for monitoring key components (sugars and acids) of fruits. However, the technique’s capacity to detect softening (textural changes) is found to be limited. A study on ladyfinger was also conducted by Shukla et al. [47] using ATR-FTIR for biochemically characterizing the different parts of ladyfinger (exocarp, mesocarp, seeds) by separating their signals. The ATR-FTIR could find peaks of protein and essential amino acids higher in the seeds than in the other parts. The novelty of this research is related to the segregation of different fractions of the crop with minimal or no sample preparation, generating a more comprehensive molecular profile. At the same time, the sample studied was from only one market, thus lacking replications, other cultivars, and multivariate calibrations.

Hassaini et al. [48] demonstrated that fig cultivars’ peel spectra with PCA can effectively differentiate chemical components, providing superior discrimination due to their higher pigment and antioxidant content. Distinct absorption bands corresponding to carbohydrates, proteins, esters, and phenols were identified, supporting their potential in breeding, genetic preservation, and authenticity certification. Similarly, ATR-FTIR could rapidly detect changes in antioxidant compounds in strawberries and raspberries under varying storage conditions. Using PCA and hierarchical cluster analysis (HCA), Sachadyn-Król et al. [49] demonstrated apparent spectral clustering linked to temperature and storage duration, particularly in regions associated with phenolics and flavonoids, with lower temperatures preserving antioxidant profiles more effectively. Likewise, strawberries have been further investigated for shelf-life monitoring. Ladika et al. [16] integrated ATR-FTIR with chemometrics, showing that deterioration during storage corresponded to changes in O–H, C–H, and C=O bands, particularly across 3645–3600 cm^−1^ (moisture), 2920–2918 cm^−1^ (sugars), and 1742–1730 cm^−1^ (organic acids). These spectral changes were accompanied by compositional variations (moisture, soluble solids, and phenolics), with PCA reliably categorizing samples by storage period. Similarly, Schorn-García et al. [19] used ATR-FTIR to monitor grape ripening, using PCA to categorize the ripening stages. At the same time, partial least squares regression (PLSR) was employed to predict soluble solids and pH accurately. Collectively, the investigation highlighted the extensive uses of ATR-FTIR with chemometrics in the analysis of fruit quality profiles, maturity, and early detection of spoilage during post-harvest storage.

**Figure 3:**
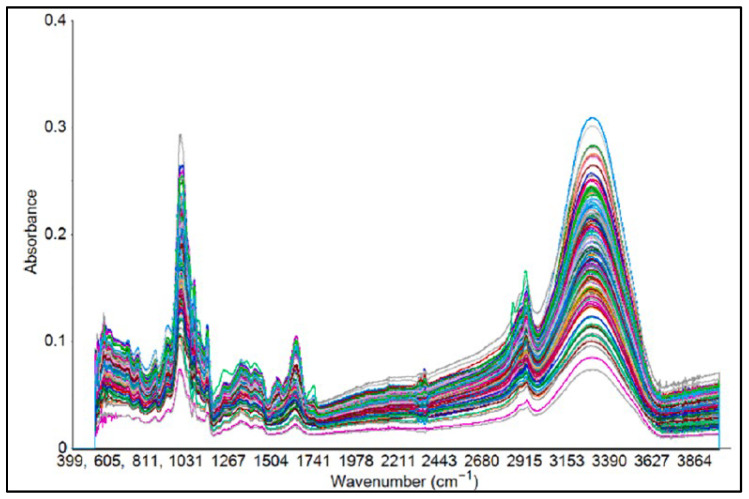
Roots (arrowroot, cassava, canna, and taro) and tuber (White, yellow, and purple sweet potato) flour samples spectra. Reproduced with permission [53].

The ATR-FTIR spectroscopy, combined with chemometrics, is increasingly used to monitor and ensure the quality and safety of vegetables, thereby ensuring consumer satisfaction. In this context, Quijano-Ortega et al. [18] investigated ATR-FTIR with PLSR to assess carotenoids in Cucurbita species. Using freeze-dried pulp and extract samples, the authors achieved high predictive accuracy (R^2^ values of 0.95 and 0.93 for calibration and prediction, respectively) in the spectral range of 950–980 cm^−1^, in which β-carotene and lutein can be detected. Likewise, Falcioni et al. [50] highlighted that chlorophylls, carotenoids, and anthocyanins were correctly detected across the fingerprint region in the ATR-FTIR spectra of lettuce using PCA-LDA and support vector machine (SVM), with an accuracy of around 97%. This characterizes the technique as a high-throughput approach for varietal differentiation in breeding and precision agriculture. ATR-FTIR has also been employed to study the impacts of soil and nutrient management on vegetables. Losacco et al. [51] applied the technique to evaluate the effects of nitrogen fertilization and biochar amendments in cauliflower, identifying spectral markers of water, proteins, carbohydrates, and lipids, including O–H (3290 cm^−1^), C–H (2930 to 2860 cm^−1^), C=O (1742 cm^−1^), and amide bands (1649 and 1540 cm^−1^). Principal component analysis aided ATR-FTIR in detecting the changes in nutritional and physiological aspects impacted by soil alterations by nearly 99.3% of the variance. Applications included post-harvest processing as well. Tepe [52] highlighted the effects of pretreatment (ethanol) and drying time on potato slices. The combination of ATR-FTIR and PCA showed that both the pretreatment and drying protocol strongly impacted the quality of potato slices, providing insights relevant for the potato industry. Additionally, Masithoh et al. [53] applied ATR-FTIR spectroscopy (4000–600 cm^−1^) with PCA and PLSR for root (arrowroot, cassava, canna, and taro) and tuber (sweet potato: white, yellow, and purple) flours, achieving predictive coefficients of almost 0.93 for protein and 0.77 for glucose. The important absorptions included CN stretching (1645 cm^−1^), CH stretching (2921 cm^−1^), and NH/OH stretching (3290 cm^−1^), validating the feasibility of the technique for accurate compositional analysis (spectra can be seen in Figure 3). Overall, these studies demonstrate the adaptability of ATR-FTIR spectroscopy in monitoring the biochemical and structural features of vegetables across various pre- and post-harvest stages. These provide high throughput to support the quality of foods as well as sustainable agricultural practices.

#### 2.2.2. Grains

Each cereal or pulse variety has a unique biochemical characteristic that affects the quality of its final uses. Lin et al. [54] emphasized the prospects of ATR-FTIR in screening sorghum, particularly for quantifying protein and tannins. The study revealed that the amide I and protein levels were strongly associated at 1652 cm^−1^, with a predicted R^2^ of 0.945. Moreover, varieties rich in tannins displayed three distinct absorption peaks (3300, 1608, and 1522 cm^−1^). This enabled the discrimination of samples with great accuracy.

Wheat is used primarily as wheat flour (whole and refined) for various end uses worldwide. During the commercial milling of wheat flour (especially refined/multipurpose wheat flour), the endosperm (which is full of starch) is separated for further size reduction. The other parts of the wheat grain (especially the germ and bran) are treated as by-products. Hadjadj et al. [55] conducted a study on the thermal stability of wheat germ, which is rich in lipids and lipase enzymes, under various temperature conditions. They found that a treatment at 80 °C for 20 min was the most effective method to inactivate the lipase enzyme and regulate the formation of free fatty acids while preserving other nutritional biochemical parameters. The ATR-FTIR revealed that there were no significant changes in the functional groups corresponding to protein and lipid. This approach to inactivating the enzyme while retaining nutritional properties is valuable. However, it can be noted that different wheat samples (varieties) may vary in their germ compositions and enzymatic activity, while pretreatments and milling processes can have other effects. Generalizing to various samples of wheat germ and industrial streams could require further optimization in conjunction with multivariate chemometrics. Meanwhile, Pro et al. [56] demonstrated the ATR-FTIR capacity to study the effects of nitrogen fertilization and soil tillage practices (agronomic management) on the leaves and caryopses of durum wheat. The spectra reflected the effects of agronomic practices on the metabolic responses, as revealed by peak shifting in the areas corresponding to macromolecular components. Srinuttrakul et al. [57] applied ATR-FTIR, along with orthogonal projections to latent structures discriminant analysis (OPLS-DA), to evaluate the Hom Mali rice from different regions of Thailand. Varieties were classified with a very high accuracy for two consecutive years of production (96.97% and 100%). These studies reflect the strength of the technique in analyzing the compositional and regional classification of several crop varieties. On a different note, Tsagkaris et al. [58] used ATR-FTIR in combination with PCA and OPLS-DA to successfully detect adulteration, correctly distinguishing between spelt and common wheat. This outcome shows that ATR-FTIR can serve as a promising tool for protecting the interests of consumers and abiding by regulatory compliance.

Apart from cereal grains, ATR-FTIR has been applied to detect the nutritional profiling of pulses and ensure their authenticity. In this regard, Biancolillo et al. [59] exhibited the effectiveness of the technique in grouping lentils of different regional origins, specifically varieties of high value (protected under “Protected Geographical Indication”, such as Castelluccio di Norcia and the “Slow Food Presidium” label, like Santo Stefano di Sessanio). Differentiation with high accuracy was enabled using chemometric models, such as partial least squares discriminant analysis (PLS-DA), thus highlighting the ability to conserve the integrity of high-value crops. In another related study, Madurapperumage et al. [60] used the same spectroscopy technique with PLS in three crops (chickpea, dry pea, and lentil) for the quantification of total protein and sulfur-containing amino acids (SCAA). The models showed R^2^ = 0.84–0.94 (total protein) and R^2^ = 0.81–0.82 (SCAA).

#### 2.2.3. Others

The application of ATR-FTIR is increasing in nearly all sectors of the food industry, including the oil and dairy industries. For oil quality analysis, Durand et al. [61] achieved consistent results using a peroxide value (PV) assay by integrating the triphenylphosphine/triphenylphosphine oxide reaction with ATR-FTIR, with minimal reagent use and a quicker analysis time. Similarly, Revelou et al. [62] differentiated olive oils based on cultivars, using a combination of ATR-FTIR with chemometrics (LDA and QDA) and SPME-GC-MS. Cultivars were classified with 97–100% accuracy. However, it is worth noting that a gap exists in environmental variability and validation across wider datasets. In the dairy industry, it remains challenging to assess the quality and safety of nutrient-rich milk due to its complex composition. Jha et al. [63] studied the reliability of ATR-FTIR in detecting aflatoxin B1 (AFB1) within the specific spectral region of 1484 to 1423 cm^−1^. When combined with PLS and multiple linear regression (MLR), the results yielded high predictive accuracy, with R^2^ values of 0.92 and 0.90 (calibration) and 0.97 and 0.92 (validation). This enables reliable monitoring of the quality and safety of milk. However, there is a need for broader validation under different storage and supply chain conditions to ensure its robustness. Taken together, these studies highlight the high performance of ATR-FTIR for quality and safety assessment in the oil and dairy industries, while also needing broader validation to heighten reliability for commercial applications.

Spices play a significant role in enhancing food flavor, also contributing to antimicrobial properties imparted by their bioactive compounds. As such, spices are considered natural preservatives. Cinnamon cultivars, popularized by their unique flavor and bioactive composition, were correctly distinguished between organic and non-organic by Bruni et al. [64] when employing ATR-FTIR. Furthermore, the Zingiberaceae rhizomes’ quality and chemical compositions were studied and characterized for eight samples. ATR-FTIR with PCA and cluster analysis (CA) successfully differentiated the samples with 96% accuracy. The study demonstrated the simultaneous application of ATR-FTIR in quality control and identification of spices [65]. However, further studies are needed on other varieties and processing conditions to confirm the technique’s potential for large-scale implementation.

### 2.3. Safety Monitoring and Adulteration Detection of Food and Agricultural Products

As per the food safety standard guidelines, threshold limits are specified for possible physical, chemical, biological agents, or allergens, if present in food, beyond which they become a health hazard. Therefore, it is crucial to meticulously detect the presence of any potential hazards in food. ATR-FTIR, coupled with chemometrics, has been employed to detect adulteration and assess the safety of food and agricultural products from microbial hazards. Several recent studies are highlighted herein.

Adulteration of oils and fats is a prohibited practice that compromises the product’s quality, safety, and cultural aspects. This demands a detection technique that is accurate, quick, and efficient. Akram et al. [66] applied ATR-FTIR spectroscopy with chemometrics to identify adulteration of vegetable oil in butter. PCA achieved differentiation accuracy of 98%, but predictive accuracy was moderate, R^2^ = 0.88 (calibration) and R^2^ = 0.68 (validation). Thus, ATR-FTIR has the potential for quick detection of butter adulteration. However, the moderate validation performance indicates that stronger models are required. ATR-FTIR has been applied widely for the validation of its authenticity and for religious conformity for edible oils. The high sensitivity of the technique was demonstrated by Windarsih et al. [67] in detecting snakehead fish oil adulteration with pork oil adulteration. PCA, OPLS-DA, and PLS achieved 99% accuracy even at adulteration levels as low as 5%, thereby showcasing their relevance for the verification of halal practices. Furthermore, Wang et al. [68] used ATR-FTIR with a transformer encoder–support vector machine regression to detect adulterants in camellia oil. This technique had the capacity to capture the variation in absorbances and identify adulteration at a minimal level. Collectively, the studies demonstrated the versatility of ATR-FTIR in detecting adulterants across a wide range of oils and fats, including those of plant and animal origins. Yet, a significant gap still exists in oils and fats from various sources, to address their compatibility in quality and safety monitoring at the commercial level.

**Figure 4:**
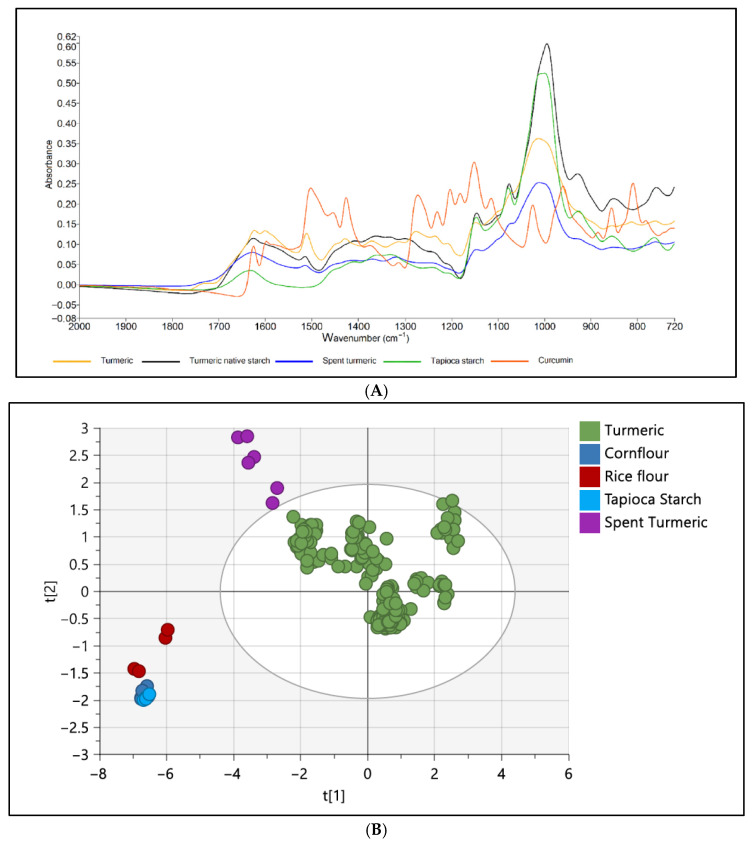
Attenuate total reflectance–Fourier transform infrared (ATR-FTIR) spectra presenting reference samples (Red: curcumin, Orange: turmeric, and Black: native turmeric starch) and adulterant samples (Blue: spent turmeric and Green: tapioca starch) (**A**). PCA chemometric model for the detection of turmeric and adulterants using FTIR (**B**). Principal components (t1 and t2). Reproduced with permission [71].

ATR-FTIR has also been successfully applied in the detection of adulterants in sugar-rich products like honey, fruit juices, and nectars. These are highly targeted products for adulteration due to their high demand and market value. Limm et al. [69] demonstrated that ATR-FTIR with chemometrics accurately detected and classified honey adulterated with syrups (corn and rice) with 88.3% accuracy. Although adequate, moderate accuracy indicates that the enhancement of models is necessary to encompass the rich compositional diversity of honey. Likewise, cheap and low-quality sugars are commonly blended with fruit juices. A study by Dhaulaniya et al. [4] successfully detected the adulteration of apple juice with cane sugar using ATR-FTIR spectroscopy. Miaw et al. [70] identified the major fruit constituents being adulterated in nectars by the application of ATR-FTIR with multivariate calibration. Shannon et al. [71] employed ATR-FTIR and chemometrics to detect the adulteration of turmeric powder. This technology, in conjunction with chemometric analysis, effectively differentiated between authentic and adulterated turmeric samples. Figure 4A illustrates the IR spectrum of both authentic turmeric and adulterated samples. The unsupervised method facilitated the identification of differences and PCA plots, as shown in Figure 4B, demonstrating a clear distinction between turmeric and its adulterants. Fattahi et al. [25] noted that saffron, a costly spice, is often adulterated with safflower, which can lead to health complications. They used ATR-FTIR spectroscopy, feature selection algorithms, and machine learning techniques to detect adulteration in saffron. The study assessed classification accuracy using PCA and SVM models. The chemical composition of the samples influenced the FTIR spectra, revealing similar levels of adulteration clustering with minimal overlap. Pure saffron samples were easily distinguished from adulterated ones; however, the adulterated samples showed some overlap, and the spectroscopic approach neglected spatial variation.

Lv et al. [72] noted that pesticides are widely used worldwide to protect crops from harmful organisms; yet, residues may infiltrate the food chain, resulting in life-threatening disorders. They conducted a study to monitor pesticide residues in tomato fruits using ATR-FTIR spectroscopy for quantitative characterization. The spectra were acquired via a diamond crystal ATR mounted to the FTIR system and examined through chemometric methods, yielding a classification model accuracy of 93.33%. Quantitative analysis was conducted on wavenumber ranges of 1800–1000 cm^−1^ and 2900–2700 cm^−1^ utilizing correlation analysis and the interval partial least squares method. The regression model created using PLSR showed exceptional performance, exhibiting an R^2^ value of 0.80 and a root mean square error (RMSE) of 1.02 μg/cm^2^. This work emphasized the capability of ATR-FTIR, integrated with chemometrics, for the rapid, in situ quantitative evaluation of pesticide residues in foods. Li et al. [73] highlighted the application of ATR-FTIR for detecting pesticide residues in apple farming. The authors noted the risks of excessive and uneven application, resulting in residues that exceed permissible limits, which endanger human health and the environment. They emphasized the need for a rapid, secure, and dependable method to identify and monitor various pesticide residues in apples. The researchers adopted ATR-FTIR spectroscopy to identify multiple pesticide residues on apple peels. A multi-task learning (MTL) model utilizing multi-task neural networks was developed for the qualitative and quantitative analysis of three pesticides, enhancing detection efficiency and practical use. The MTL model achieved 100% accuracy in differentiating three types of pesticide residues and demonstrated high-precision predictions in quantitative tasks, with R^2^ and RMSE values of 0.94 and 2.57 μg/cm^2^, respectively. This provided a real-time detection of pesticide residues in complex environments.

Zhou et al. [74] discussed recent advances in molecular vibrational spectroscopy, focusing mainly on the mid-IR (ATR-FTIR) for detecting foodborne pathogens, as well as the role of chemometrics and artificial intelligence (AI). The conclusion drawn from this investigation is that ATR-FTIR can discriminate microbial strains using chemometrics (PCA, PLS-DA, SVM) and spatial markers, such as protein (Amide I/II), lipids, and polysaccharides. However, ATR-FTIR works better for low-moisture materials. Shortcomings include the limited performance of real-matrix and calibration transfer. Microorganisms are used in both traditional and industrial food fermentation. In the context of winemaking, yeast (*Saccharomyces cerevisiae*) ferments carbohydrates to alcohol. In this process, the cell wall components of the strains matter. Binati et al. [75] studied the cell wall component of *Saccharomyces cerevisiae* using ATR-FTIR microspectroscopy and adopted PCA for analysis of the spectral data. Differences among the strains in the polysaccharides and β-glucans in intact cells and separated cell walls were observed. The authors claimed that this technique applies to intact cells without much invasive treatment for selecting the desired cell walls of yeast strains. However, since the experimental strains were grown under laboratory environments, results might differ under actual winemaking conditions.

According to the available literature, it is expected that ATR-FTIR, coupled with chemometrics, will serve as a powerful technique for detecting the molecular characteristics of food and agricultural products, facilitating both qualitative and quantitative analysis, as well as the detection of adulteration for safety and quality assurance. Table 1 summarizes the ATR-FTIR features and chemometrics for the applications on quality and safety studies of food and agricultural products.

## 3. Synchrotron Radiation

A synchrotron is a high-energy electron storage ring designed to generate and gather intense light emitted by electrons during acceleration [37]. Synchrotron radiation (SR) functions as a powerful tool for scientific research, characterized by features such as high intensity, broad spectrum range, narrow angular collimation, high polarization, pulsed timing, and exceptional brightness. It enables detailed studies of materials in small quantities and is suitable for various spectroscopic techniques. The radiation’s small cross-sectional area and tight collimation increase its brightness. Experiments with synchrotron radiation are conducted in ultra-high-vacuum conditions, ensuring excellent beam stability and repeatability. These features can be quantitatively evaluated, allowing researchers to customize the radiation for specific experimental needs and interpret results precisely [76]. When particles with similar charges are accelerated perpendicular to their motion, they produce electromagnetic radiation known as SR. Accelerated electrons move near the speed of light (*c*), with their velocity (*v*) ratio represented as *v*/*c* = *β* = 0.99999. The Lorentz factor *γ* measures an electron’s relativistic effects, expressed through particle velocity and light speed, or as the ratio of total energy (*E*) to rest mass energy (*m_o_c*^2^), as shown in Equation (5) [77].

(5) $$\gamma =\sqrt{1-\frac{v^{2}}{c^{2}}}=\sqrt{1-\beta^{2}}=\frac{E}{m_{o}c^{2}}=\frac{E\;(GeV)}{0.511\;(MeV)}=1957E(GeV)$$

**Figure 5:**
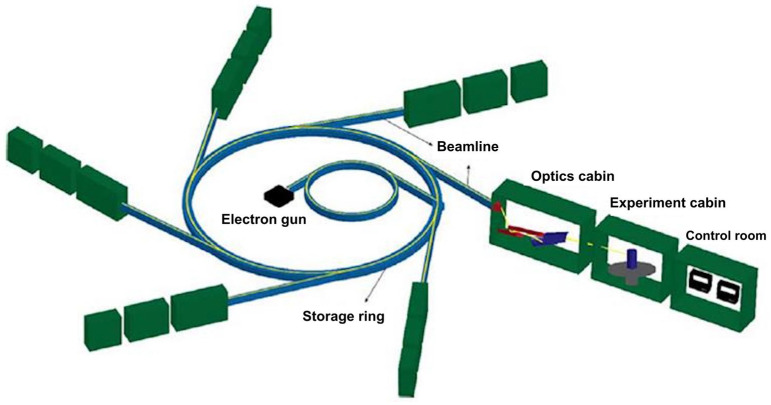
Main elements of a beamline system from synchrotron radiation. Reproduced with permission [79].

The generated frequencies can span a significant portion of the electromagnetic spectrum. Unlike traditional sources that emit radiation from a large area, synchrotron radiation sources produce radiation in a narrow cone, following the path of fast-moving electrons [77]. After generation and acceleration in the injection phase, electrons are injected into the main ring and can travel at nearly the speed of light for hours or days [78]. A typical SR facility, as shown in Figure 5, includes six major components [79] (an electron gun and linear acceleration) for initial acceleration, a storage ring for holding electrons, a beamline, optical components like slits, filters, and mirrors, a sample chamber for experiments, and a control room for data collection and analysis. The electrons are first accelerated by a linear accelerator (LINAC) to attain millions of electron volts (MeV), then boosted to giga-electron volts (GeV) before reaching the main storage ring [76]. This setup produces extremely high-intensity infrared (IR) radiation that exceeds that of traditional sources. The electron beam in the storage ring emits radiation as it passes through bending magnets, undulators, and wigglers. Radiation is emitted as electrons pass through the space between the magnetic fields of the bending magnets. A wiggler produces a beam with a continuous spectrum, like a bending magnet, but with much higher intensity. An undulator’s dipole magnet arrangement creates interference patterns, resulting in multiple bright spectral bands with high intensity [80]. Bhuiyan et al. [37] noted that synchrotron light was intense and precise, with IR light being 100 to 1000 times brighter than conventional thermal sources. It can cover a broad infrared wavelength range, from NIR to far-IR, with sharper peaks in the MIR region. The MIR spectra offer better resolution than near-IR spectra, enabling the identification of specific chemical groups within mid-IR bands. Tanino et al. [81] added that mid-IR light can detect and analyze chemical substances in plant tissues, bulk tissues, or segments. When paired with appropriate detectors or microscopes, FTIR can image at scales as small as 2 μm, revealing differences in cell wall composition and providing a higher signal-to-noise ratio [82].

### 3.1. Synchrotron (SR)-Based FTIR Spectrophotometer

**Figure 6:**
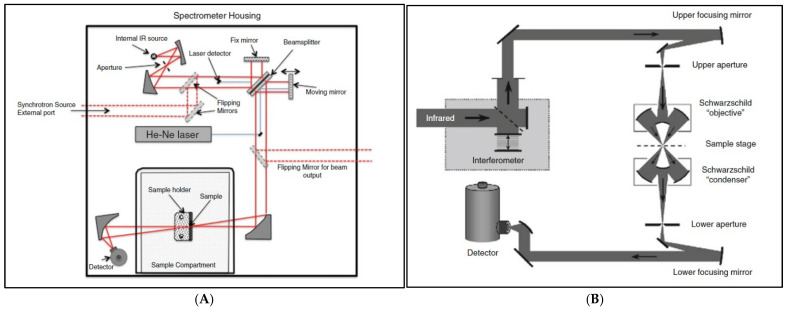
(**A**) Synchrotron-based Fourier transform infrared (FTIR) microspectrometer; (**B**) FTIR instrument integrated with a microscope and an infrared detector. Reproduced with permission [83].

Dumas et al. [83] and Bhuiyan et al. [37] explained that SR-FTIR spectroscopy involves the interference of radiation between two beams to produce an interferogram, which is a signal based on the change in path length between the two beams. The two domains of distance and frequency are interconvertible using the Fourier transformation. The radiation from the synchrotron source is typically passed through an interferometer, where a beam splitter separates the incoming beam into two parts: one directed at a fixed mirror and the other at a moving mirror. The interferometer then recombines the two beams. The combined beam is directed to the sample and a detector. The data are converted to digital form by an analog-to-digital converter and transferred to a computer for Fourier transformation (Figure 6A). The IR microspectrometer is an FTIR instrument integrated with a microscope and an infrared detector, enabling spectroscopic analysis of a localized region (Figure 6B). It captures point spectra and produces chemical images via raster scanning. A single-element detector is used, and reflecting Schwarzschild-type objectives are employed to minimize chromatic distortions. An aperture can be positioned at one or both axial foci, limiting the lighted or detected region on the specimen. An ATR accessory can be integrated into the SR-FTIR system. In this case, samples are in direct contact with a high refractive index crystal. In fact, integrating the ATR-FTIR with synchrotron-based (SR-ATR-FTIR) microspectroscopy has been shown to improve absorption measurement accuracy, with spatial resolutions up to four times higher [84].

### 3.2. Applications Using Synchrotron Technology

Synchrotron techniques can be widely applied to the agricultural and food sectors, with uses including crop disease monitoring, detection of grain development stages, compositional analysis, and evaluation of the molecular structure of food matrices, among others. This section provides a more detailed description of these applications by discussing recent studies.

#### 3.2.1. Quality of Food and Agricultural Products

The synchrotron-based FTIR approach facilitates the relationship of spectral data with the structural and biochemical characteristics of plant and seed tissues, as well as the quality of feed and food. Phansak et al. [85] noted that rice remains one of the most vital cereal crops globally, serving as a staple food, particularly in Asia. However, rice is susceptible to several diseases. Rice blast, a fungal disease, remains a threat to rice production (caused by *Magnaporthe grisea*). The deployment of resistant rice cultivars has proven to be an effective and environmentally sustainable strategy for managing this disease. Considering this, recent advancements in spectroscopic techniques have opened new avenues for screening and identifying blast-resistant rice varieties. Furthermore, to enhance the precision and efficiency of such screening methods, Phansak et al. [85] investigated the use of SR-FTIR microspectroscopy for screening and identifying blast resistance of 80 rice cultivars and two reference lines 14 days post-inoculation with the blast pathogen. Spectral data for the samples, i.e., susceptible (SUS), moderately susceptible (MOD), tolerant (TOL), sensitive (SENT), and reference (RES), were acquired. The spectra underwent chemometric studies (PCA and HCA) to augment spectral characteristics. Thus, SR-FTIR can be considered as an innovative offering with the promising capacity to analyze and differentiate what the conventional techniques fail to achieve.

The SR-based FTIR techniques have been exhibiting their potential for the quality and safety assessment of crops as an advanced, minimally destructive approach. SR-FTIR has been employed by Ranathunga et al. [86] to study rice grain at three different stages of development (milky, dough, and mature). The different components of rice grain (pericarp, aleurone, endosperm, and air cavities) were categorized accurately using PCA and HCA. It can be noted that conventional techniques often find it difficult to analyze precisely the structural and molecular changes as SR-FTIR does. This emphasizes SR-FTIR uniqueness for categorization and analysis of grain nutritional profiling. Kongman et al. [87] noted that Thai jasmine rice is popular on the global market due to its distinctive attributes, including aroma, fragrance, flavor, shape, size, and texture, resulting in high consumer demand. However, due to its sensitivity to photoperiod and inadequate disease resistance, its annual crop output is limited. SR-FTIR has been used to analyze the biochemical composition of the improved mutant rice characteristics. PCA was used to analyze the spectra to differentiate the rice traits based on their carbohydrate and protein bands. The findings revealed the capability of SR-FTIR to differentiate various Thai jasmine characteristics based on their biochemical constituents. Comparable research may be conducted to analyze the nutritional content of the crop. Wheat is another cereal grain playing a significant role both as food and feed worldwide. Indore et al. [88] noted that wheat grains encounter challenges during bulk storage. Xin et al. [89] noted that wheat is a primary crop in Western Canada’s grain export sector and is widely consumed due to its protein content. In some years, the crop suffers 50% late maturity damage owing to the prevalent hard frost in the area. As a result, it compromises the quality of flour and its baking properties, rendering it unfit for human consumption. Consequently, Xin et al. [89] performed research using SR-FTIR to analyze the protein structure (amide I, II, and secondary structures), carbohydrate structure, and functional groups in normal vs. frost-damaged wheat. The SR-FTIR provided the molecular microstructural attributes of the wheat tissues. These results would enhance the comprehension of frost-damaged grains and the associated industry.

The above studies of SR-FTIR spectroscopy have proven its potential for disease management and nutritional profiling of crops and plants. Additionally, its application for the effective discrimination between salicylic acid-ricemate-treated and non-treated rice for the bacterial leaf blight using PCA was demonstrated by Thepbandit et al. [90]. Beyond disease detection, SR-FTIR has also been applied to crops of nutritional and commercial relevance. Dorion and Yu [91] characterized the flaxseed protein structures, revealing treatment-induced changes in the α-helix to β-helix ratios and demonstrating enhanced sensitivity when focusing on the amide I and II bands. These studies collectively point towards SR-FTIR, combined with chemometrics, as a powerful, minimally destructive technique for investigating crop disease, composition, structure, and the effects of processing, with comprehensive applications in the food sector [92,93,94]. Complementing this, Rodriguez-Espinosa [95] also studied faba bean endosperms and demonstrated alterations in the cellular and molecular structures of proteins resulting from pressure toasting (steam). The results provided comprehensive insights into the chemical structural characteristics and cellular dimensions of plant tissues.

In agricultural practices, seed quality is essential for its viability, high yield, and resistance against stresses [96]. The SR-FTIR microspectroscopy has been a promising minimally destructive technique for the analysis of seed molecular and cellular resolution. Regarding this, Liu and Yu [97] recognized variations in the composition of endosperm, particularly among barley cultivars. Correspondingly, Yu et al. [98] revealed comparable protein amide I structures in yellow and brown canola seeds and exhibited differences in their relative protein proportions. Thus, it can be understood that SR-FTIR enables the exploration of compositional and structural dimensions in grains and seeds, offering opportunities for targeted breeding and nutritional profiling. Nevertheless, validation and standardization for the broader implementation of SR-FTIR are still required.

The SR-FTIR applications have also extended to non-grain foods such as dairy, meat, and their related products. Alteration in the secondary protein structures of tilapia fish by sous vide cooking has been shown by Pongsetkul et al. [99]. Clearly stating the β-sheet as the primary determinant of meat texture. At the same time, transitions in the structure with respect to cooking time and temperature were effectively analyzed using PCA, thus exhibiting the effects of heat on the quality of meat. Another study by Charoensin et al. [100] characterized the chicken muscle fibres and protein structure from the spectra, recognizing peaks at 1645 cm^−1^ (α-helix), 1685 cm^−1^ (β-turn), and 2927 cm^−1^ (CH_2_ asymmetric stretching). Findings from these studies highlight SR-FTIR, with chemometrics a promising approach for chemical profiling of meat, particularly protein. Such an approach can also serve as a positive strategy in breeding poultry.

In the context of the dairy sector, Pax et al. [84] investigated the mapping of secondary protein structures in mozzarella cheese using SR-FTIR microspectroscopy with HCA and PCA, which enabled the accurate characterization of compositional variability in the samples. This demonstrated the technique’s utility in monitoring structural changes that influence product quality and in supporting dairy innovation. Meanwhile, Ong et al. [101] investigated the effect of pH on the quality parameters of cream cheese using SR-ATR-FTIR microspectroscopy. The study could link the molecular secondary structures (β-turn and β-sheet) with the microstructure, texture, and rheological properties of cream cheese, thus exhibiting the technique’s strength and robustness in a multi-scale comprehensive approach. In another study, Pax et al. [84] examined the interaction between protein, fat, and water at the microscale and its influence on the texture and overall quality of mozzarella cheese. The researchers achieved this goal using SR-FTIR microspectroscopy, coupled with an ATR accessory, and multivariate chemometrics (HCA and PCA). The study revealed protein and fat globules in a heterogeneous matrix with its spatial distribution gradients. Protein interaction with lipids is critical to cheese meltability and texture. The SR-FTIR technique conducted in ATR measurement mode, along with chemometrics, created a multiscale view revealing microscale molecular architecture and bulk composition.

Kim et al. [39] used SR-FTIR (with ATR mode) to examine the surface composition and encapsulation of β-Carotene, a bioactive compound sensitive to oxygen, light, and heat, in spray-dried microcapsules using blends of pea and whey protein as wall materials. The study gave a high-resolution microstructural mapping of the micro-particle surface with the potential for modelling a chemometrics approach. At the same time, some limitations were detected regarding the spatial penetration depth of ATR-microspectroscopy, which is on the order of microns or less. Therefore, the internal or deeper core distribution study may not be reflected, as it is informative for the surface oil but cannot capture the entire internal structure. Also, additional calibration or chemometric models would be needed to transform the IR intensity into concentration. Chia seed oil is rich in omega-3 and is highly prone to oxidation; hence, encapsulation is crucial. Traditional analysis can provide compositional information, but not the spatial interaction between protein, lipid, and carbohydrate within a single microcapsule. Timilsena et al. [102] used SR-ATR-FTIR microspectroscopy to facilitate chemical mapping of single particles for the identification of the spatial distribution of chia seed oil (spray-dried microcapsules). They also applied HCA to understand the spectral data. Thus, SR-ATR-FTIR microspectroscopy demonstrated its potential to provide an understanding of structure and functional relationships.

Bouchon et al. [103] examined the absorption of oil in fried potatoes using SR-FTIR. The approach can differentiate patterns of absorption between the crust and the core of fried potatoes. The results validate the robustness of SR-FTIR with chemometrics for characterization of structural and compositional changes within dairy and fried foods. Conversely, the need for specialized synchrotron facilities remains a limitation for extensive applications in industry. However, practical limitations remain a constraint with expensive facilities and accessibility restrictions of synchrotron sources.

#### 3.2.2. Safety of Food and Agricultural Products

In the food industry, microorganisms can play a dual role, being essential for fermented products or serving as agents of food spoilage and food-borne illnesses (pathogenic microorganisms). Foodborne diseases and illnesses (food intoxication and food infection) are caused by the consumption of food contaminated with microorganisms, bacteria, viruses, parasites (*Salmonella* spp., *Campylobacter jejuni*, *Shigella* spp., *L. monocytogenes*, *Clostridium perfringens*, *Enterotoxigenic E. coli*, and *Vibrio cholerae*), mycotoxins, chemicals, or other pathogenic agents [104,105]. Wang et al. [106] conducted a study on the discrimination of foodborne disease-causing bacteria using SR-FTIR microscopy and chemometric analysis (PCA). The results demonstrated that spectral regions of macrocomponents, 3000–2800 cm^−1^ (lipids), 1800–1500 cm^−1^ (proteins), and 1200–900 cm^−1^ (polysaccharides), discriminated bacteria using SR-FTIR microspectroscopy effectively; the SR-FTIR method was confirmed to be a powerful approach for the classification of microbial at different levels (species and subspecies). Application of this approach is not limited to bacteria but also extended to Mycotoxin (aflatoxins, patulin, ochratoxin A) producing fungi, including *aspergillus* spp. and *penicillium* [107]. One such fungus, which produces mycotoxin and is difficult to identify, black aspergillus, represents a challenge worldwide [107,108]. Advanced SR-FTIR, in combination with deep learning, improved its differentiation at different levels with high accuracy greater than 95%, over the spectral range of 3700–800 cm^−1^ [109]. Likewise, Lu et al. [110] and Sukprasert et al. [111] further detected *A. flavus* infection and aflatoxin B1 accumulation in maize in the wavenumbers ranging between 1733 cm^−1^ and 1361 cm^−1^, and Salmonella was detected, exhibiting a distinct peak using SR-FTIR with ferromagnetic nanoparticles.

Analyzing the live cell composition and characteristics of microorganisms is both complex and crucial. In this context, Meneghel et al. [112] customized an SR-ATR-FTIR microspectroscopy and used it to probe the composition of live bacteria in an aqueous medium. They observed that it could allow discrimination of the strains and heterogeneity in the bacterial populations. Also, the study showed that cryo-sensitive cells exhibited higher protein content, exhibiting an α-helix structure in the studied spectral region. This outcome showcased that SR-ATR-FTIR microspectroscopy, powered by its high resolution, can aid in the detection of live cells in an aqueous medium to understand single cells, clusters, and heterogeneity. Nevertheless, this type of analysis can suffer from the interference of artifacts from water, as even small amounts may be strongly absorbed in mid-IR. Also, overlapping of bacterial molecules (polysaccharides, lipids, and proteins) may occur.

Aside from these advantages, there are also significant limitations. The studies were confined mainly to laboratory settings and could not be applied to the monitoring of food in realistic industrial scenarios. Scalability and the reproducibility of the complex food matrices and biodiversity remain unexplored. The existing gaps highlight the need for further research to translate SR-FTIR from laboratory trials into robust, real-time, field monitoring for pathogens and food safety. The above-discussed SR-FTIR features and chemometrics for the applications in quality and safety studies of food and agricultural products are summarized in Table 2.

## 4. Techniques (ATR-FTIR and SR-FTIR) at a Glance

As emerging technological tools for crop and food analysis, ATR-FTIR and SR-based FTIR are constantly evolving. Currently, both technologies are mainly restricted to the research ecosystem, where new features and applications are being developed. As such, these minimally destructive techniques still face drawbacks regarding their large-scale implementation, although notable advantages have been extensively described in the literature. Additionally, Table 3 summarizes the key features of ATR-FTIR and SR-based FTIR.

## 5. Conclusions

The quality and safety of food and agricultural products, and their evaluation, are of paramount importance. The use of conventional analytical methods, including wet chemical analysis, sensory evaluation, and textural assessment, can be time-consuming, environmentally harmful, and sometimes unreliable. Mid-infrared (MIR) spectroscopy, specifically Fourier transform infrared (FTIR), used under attenuated total reflectance (ATR) mode, as well as synchrotron-based Fourier transform infrared (SR-FTIR) with or without the ATR measurement mode, represents an alternative to traditional analytical protocols for assessing food safety and quality. FTIR is a rapid, versatile, chemical-free, eco-friendly, and minimally destructive methodology that provides comprehensive insights into the diverse quality characteristics of food and agricultural products, including physicochemical, nutritional, textural, and microbiological properties, as well as the identification of adulterants and contaminants through complex spectral data.

The use of FTIR is diverse, encompassing all sectors of the food and agricultural products industry, including fruits, vegetables, grains, legumes, oilseeds, dairy, meat, and other products, in both raw and processed forms. The integration of advanced chemometric techniques can enhance the classification of samples according to their quality attributes, varietal authentication, and geographical origin. The FTIR application is safe and non-toxic to all individuals involved in such activities, as it does not involve any harmful radiation source (γ-rays and X-rays).

The ATR-FTIR with a globar infrared source and synchrotron-based FTIR are being developed as instruments for ongoing, real-time quality assurance throughout the agri-food supply chain, extending beyond the confines of laboratories. Portable devices can monitor deterioration, contamination, and spoilage during post-harvest handling, from field to transit and storage, thereby minimizing losses and ensuring quality and safety across the supply chain. Advancements in the Internet of Things (IoT) and artificial intelligence (AI) facilitate remote analysis and decision-making. Consequently, FTIR can potentially transition from a static laboratory setting to a real-time assessment of quality and safety. Nonetheless, these systems face challenges, as SR-based FTIRs are confined to highly specialized facilities, while ATR-FTIR using a globar infrared source exhibits reduced sensitivity for micro-resolution. Variability in environmental parameters (field and storage) and sample characteristics (cultivars, provenance) can undermine model robustness. Also, high operational costs and the need for trained personnel constrain the extensive use of SR-based FTIR.

## Figures and Tables

**Table 1:** Summary of the attenuated total reflectance–Fourier transform infrared (ATR-FTIR) features and chemometrics for the applications in quality and safety studies of food and agricultural products.

Target	Category	Sample	Purpose	IR Range (cm^−1^)	ATR Crystal Type	Number of Scans	Resolution (cm^−1^)	Chemometric	Reference
Quality	Fruits and vegetables	Apricot fruit slurries	Quantification of sugars (sucrose, glucose, fructose) and organic acids (malic, citric)	4000–650	ZnSe	32	4	PLSR	[42]
		Fresh fruits and vegetables	Primary components of cell walls	4000–650	-	16	-	PLS	[44]
		Tomatoes	Soluble sugars in tomatoes	4000–600	ZnSe	32	2	PLSR and PCA	[17]
		Tomatoes	Flavor assessment	1800–800	-	-	-	PLS-DA	[46]
		Figs fruit	Antioxidant activity	4000–450	Germanium	128	4	PCA	[48]
		Strawberry and raspberry fruits	Changes in antioxidant compounds	4500–500	ZnSe	30	4	PCA and HCA	[49]
		Strawberries	Shelf life and quality deterioration	-	-	-	-	PCA	[16]
		Grape berries	Variability in grape ripening characteristics	4000–650	Diamond	32	8	PCA and PLSR	[19]
		Cucurbita (squash and pumpkin)	Total carotenoid content	4000–450	Diamond	24	4	PLSR	[18]
		Lettuce leaf	Pigment content	4000–400	Diamond	300	4	PCA and LDA	[50]
		Cauliflower	Biochemical effects of nitrogen fertilizer levels and biochar	4000–400	Diamond	32	4	PCA	[51]
		Potatoes	Impact of ethanol pretreatment and drying time on moisture removal behavior and quality parameters (color, shrinkage, total phenolic content, and antioxidant activity)	4000–400	ZnSe	-	2	PCA	[52]
		Tuber and roots (arrowroot, canna, taro, cassava, white, yellow, and purple sweet potato) flours	Protein and glucose	4000–400	-	32	3	PCA and PLSR	[53]
	Grains	Sorghum	Grain composition (protein and tannin contents)	4000–400	Diamond	-	4	Pearson’s correlation Analyses	[54]
		Durum wheat leaves and caryopses (grain)	Nitrogen fertilization levels on the macromolecular composition	4000–650	ZnSe	64	4	PCA	[56]
		Hom Mali rice	Regional discrimination	4000–450	-	6	1	OPLS-DA	[57]
Al.		Spelt	Authenticity assessment	4000–400	-	64	2	PCA and OPLS-DA	[58]
		Lentils	Discrimination of place of origin	4000–400	Diamond	10	4	PCA	[59]
		Pulses (chickpea, dry pea, and lentil)	Protein quality (sulfur-containing amino acids concentration)	4000–650	Diamond	100 (for lentil) 64 (for chickpea and dry pea)	2 (for lentil) 4 (for chickpea and dry pea)	PLS	[60]
	Others	Oil	Protocol for measurement of peroxide value	4000–400	-	16 (proposed)	4 (proposed)	-	[61]
		Olive oil	Botanical origin discrimination	4000–400	ZnSe	100	4	LDA and QDA	[62]
		Organic cinnamon	Evaluation of organic cinnamon from non-organic	4000–500	-	32	4	PARAFAC	[64]
		Zingiberaceae rhizomes	Differentiation of Zingiberaceae rhizomes	4000–650	Diamond	-	-	PCA and CA	[65]
Safety	Lipid-rich foods	Butter	Detection of butter adulteration with vegetable oil	4000–800	ZnSe	16	4	PCA and PLSR	[66]
		Snakefish oil	Rapid identification of pork oil adulteration in snakehead fish oil	4000–650	-	32	8	PCA and OPLS-DA	[67]
		Camellia oil	Detection of edible oil adulteration in camellia oil	4000–650	ZnSe	32	4	Vector machine regression	[68]
		Honey	Detection of honey adulteration with syrup or invert sugar in particular	4000–650	Diamond	128	4	PLS and PCA	[69]
		Apple juices	Detection of adulteration of apple juices with cane sugar	4000–400	ZnSe	32	4	PCA	[4]
		Nectars	Identification of main fruits in adulterated nectar	4000–650	ZnSe	16	4	PLS	[70]
		Turmeric powder	Detection of adulterants in turmeric powder	4000–550	Diamond	32	4	PCA, OPLS-DA and PLS-DA	[71]

**Table 2:** Summary of the synchrotron-based Fourier transform infrared (SR-FTIR) features and chemometrics for the assessment of quality and safety of food and agricultural products.

Target	Area of Study	Purpose	IR Range (cm^−1^)	Number of Scans	Resolution (cm^−1^)	Microspectroscopy Aperture Size/Pixel Size	SR-FTIR Location	Chemometric	Reference
Quality	Rice	Screening and identification of blast-resistant rice cultivars	4000–600	64	4	10 × 10 λm^2^	SLRI, Nakhon Ratchasima, Thailand	PCA and HCA	[85]
	Rice	Biochemical and functional structural changes during developmental stages (milky, dough, and mature)	4000–800	64	6	10 × 10 λm^2^	SLRI, Nakhon Ratchasima, Thailand	PCA and HCA	[86]
	Rice	Biochemical composition of the improved (ion-beam-induced) mutant Thai jasmine rice	4000–800	64	4	20 × 20 λm^2^	SLRI, Nakhon Ratchasima, Thailand	PCA	[87]
	Rice	Control of leaf blight infection in rice by salicylic acid-ricemate treatment	-	64	4	10 × 10 µm	SLRI, Nakhon Ratchasima, Thailand		[90]
	Wheat	Protein structure (amide I, II, and secondary structures), carbohydrate structure, and functional groups in normal vs. frost-damaged wheat	4000–800	256	4	10 × 10 µm	NSLS, New York, NY, USA	PCA	[89]
	Flaxseed	Molecular and protein structural characterization in flaxseed (cultivar: Vimy)	4000–800	128	4	10 × 10 λm^2^	NSLS, New York, NY, USA	PCA and HCA	[91]
	Faba bean	Intrinsic molecular structural characterization of faba bean seed endosperms influenced by pressure toasting (steam)	4000–750	64	4	10 × 10 µm	ALS, Berkeley, CA, USA	MIXED of SAS 9.4 software	[95]
	Barley	Biochemical structure of barley cultivars	4000–800	128	4	10 × 10 µm	NSLS, New York, NY, USA	MIXED procedure of SAS 9.1.3	[97]
	Canola	Molecular structures of plant proteins in the yellow and brown canola seed tissues	4000–800	64	4	10 × 10 µm	NSLS in New York, NY, USA	PCA	[98]
	Fish	Impact of sous vide cooking parameters on the physicochemical, textural, protein structure degradation, and sensory qualities of tilapia fillets	4000–400	64	4	-	-	PCA	[99]
	Chicken	Muscle fibre properties and secondary protein structures	4000–800	64	6	10 × 10 µm	SLRI, Thailand	Savitzky-Golay method in the Unscrambler X software (version 10.1)	[100]
	Cheese	Characterisation of proteins, lipids, and microstructures of mozzarella cheese	3800–700	16	4	60 × 60 µm	Australian Synchrotron Infrared Microspectroscopy (IRM), Clayton, Australia	PCA and HCA	[84]
	Fried potatoes	Oil absorption	8000–800			24 × 24 µm	Synchrotron Radiation Source in Daresbury, UK		[103]
Safety	Foodborne disease	Discrimination of foodborne disease-causing bacteria	4000–650	64	4	20 × 20 μm^2^	SSRF, Shanghai, China	PCA	[106]
	Mycotoxins	Quick identification of *Aspergillus* species	4000–400	64	6	10 × 10 µm^2^	SLRI, Thailand	1D-CNN	[108]
	Mycotoxins	Spatial and chemical changes in maize kernels infected with *A. flavus*	4000–400	64	4	20 × 20 μm^2^	SSRF, Shanghai, China	PCA	[110]
	Salmonella	Detection of Salmonella in food	4000–800	64	4	20 × 20 µm^2^	SLRI, Thailand	-	[111]

**Table 3:** Summarization of some of the main features of ATR-FTIR and SR-based FTIR.

Mid-IR Spectroscopy	Mid-Infrared Source	Interaction Mode	Depth of Penetration	Signal-to-Noise Ratio	Instrumentation	Applications
ATR-FTIR	Globar, a conventional thermal infrared Low brightness	Total internal reflection via ATR crystal (diamond, ZnSe, Ge)	Shallow (micron)	Good Limitations in detecting traces	Compact bench-top Portable Commonly accessible	Bulk sample qualitative and quantitative analysis Functional group identification, and Monitoring of chemical modification
SR-Based FTIR (With and without ATR)	Synchrotron radiation Very brilliant and Highly collimated 100 to 1000 times brighter than a conventional mid-infrared source	Transmission or reflection mode	Able to penetrate thicker materials	Extremely high Identifies analytes with low concentrations and minimal quantities (down to 1–3 µm in mid-infrared microspectroscopy)	Large-scale synchrotron facilities Expensive infrastructure	High-resolution mapping of complex and heterogeneous materials Micro-domain analysis in biological tissues, and Advanced research

## Data Availability

No new data were created or analyzed in this study. Data sharing is not applicable to this article.

## Abbreviations

1D-CNN	One-Dimensional Convolutional Neural Network
AFB1	Aflatoxin B1
AI	Artificial Intelligence
AIS	Alcohol-Insoluble Solids
ALS	Advanced Light Source
ATR	Attenuated Total Reflectance
CA	Cluster Analysis
FAO	Food and Agriculture Organization
FTIR	Fourier Transform Infrared
GeV	Giga-Electron Volts
HCA	Hierarchical Cluster Analysis
IoT	Internet of Things
IR	Infrared
IRE	Internal Reflection Element
IRM	Infrared Microspectroscopy
LDA	Linear Discriminant Analysis
LINAC	Linear Accelerator
MeV	Millions of Electron Volts
MIR	Mid-Infrared
MLR	Multiple Linear Regression
MOD	Moderately Susceptible
MTL	Multi-Task Learning
NIR	Near-Infrared
NSLS	National Synchrotron Light Source
OPLS-DA	Orthogonal Projections to Latent Structures Discriminant Analysis
PCA	Principal Component Analysis
PCA-LDA	Principal Component Analysis–Linear Discriminant Analysis
PGA	Potato Growers of Alberta
PLS	Partial Least Squares
PLS-DA	Partial Least Squares–Discriminant Analysis
PLSR	Partial Least Squares Regression
QDA	Quadratic Discriminant Analysis
RDAR	Results Driven Agricultural Research
RES	Reference
RMSE	Root Mean Square Error
SAS	Statistical Analysis System
SCAA	Sulfur-Containing Amino Acids
SENT	Sensitive
SIA	Sequential Injection Analysis
SLRI	Synchrotron Light Research Institute
SNR	Signal-to-Noise Ratio
SPME-GC-MS	Solid-Phase Microextraction Technique–Gas Chromatography–Mass Spectrometry
SR	Synchrotron Radiation
SSRF	Shanghai Synchrotron Radiation Facility
SUS	Susceptible
SVM	Support Vector Machine
SWIR	Short-Wave Infrared
TOL	Tolerant
UK	United Kingdom
USA	United States of America
